# Evaluation of adalimumab effects on left ventricle performance by echocardiography indexes among patients with immunosuppressant refractory ulcerative colitis

**DOI:** 10.3389/fmed.2022.1008711

**Published:** 2023-01-06

**Authors:** Mohammad Reza Hatamnejad, Mersedeh Karvandi, Fateme Jodatfar, Nastaran Ebrahimi, Fatemeh Shojaeian, Shaghayegh Baradaran Ghavami, Hedieh Balaii, Mahdi Moeeni, Mohsen Rajabnia, Shabnam Shahrokh, Hamid Asadzadeh Aghdaei

**Affiliations:** ^1^Department of Basic and Molecular Epidemiology of Gastrointestinal Disorders, Research Institute for Gastroenterology and Liver Diseases, Shahid Beheshti University of Medical Sciences, Tehran, Iran; ^2^Department of Cardiovascular Imaging Research, Taleghani Hospital, Shahid Beheshti University of Medical Sciences, Tehran, Iran; ^3^Department of Cardiology, Taleghani Hospital, Shahid Beheshti University of Medical Sciences, Tehran, Iran; ^4^Faculty of Medicine, Shahid Beheshti University of Medical Sciences, Tehran, Iran; ^5^Department of Surgical Pathology, Sol Goldman Pancreatic Cancer Research Center, Johns Hopkins University School of Medicine, Baltimore, MD, United States; ^6^Department of Cardiology, Seyed-ol Shohada Hospital, Urmia University of Medical Sciences, Urmia, Iran; ^7^Non-Communicable Disease Research Center, Alborz University of Medical Sciences, Karaj, Iran

**Keywords:** adalimumab, longitudinal global strain, ulcerative colitis, anti-TNF-α, echocardiography, heart failure, inflammatory bowel disease, vascular stiffness

## Abstract

**Background and aims:**

Inflammatory bases lead to a simultaneous flourishing of cardiovascular complications with inflammatory bowel disease (IBD). As a released cytokine, tumor necrosis factor-α (TNF-α) can either disrupt or preserve cardiovascular performance. Due to this controversy, this study aimed to appraise the short-term anti-TNF (adalimumab [ADA]) relics on cardiac function by gauging the echocardiography indexes in patients with immunosuppressant refractory ulcerative colitis (UC).

**Methods:**

All cases with a definite diagnosis of UC were included based on providing written informed consent and owning the severe form of active disease (Mayo score ≥7), which did not dampen with immunosuppressant. Patients were excluded in the case of previous cardiac ailments/risk factors and prior related surgical or pharmaceutical intervention. Transthoracic echocardiography (TTE) was carried out before and 3 months after biological regimen allocation and changes in indexes [ejection fraction (EF), left ventricular end-diastolic volume (LVEDV)/left ventricular end-systolic volume (LVESV), and global longitudinal strain (GLS) in standard parasternal short axis from mid-ventricular level, two-, three-, and four-chamber apical long axes] were compared *via* statistical analyses.

**Results:**

The study consisted of 13 (65%) men and 7 (35%) women, with a mean age of 36.54 ± 11.3 years. Participants mainly possessed Montreal class I (45%) and an average of 3.25 years of disease duration. The intervention significantly controlled inflammation [endoscopic Mayo score (*P* = 0.001), partial Mayo score (*P* = 0.001), and C-reactive protein (*P* = 0.001)]. Endoscopic and clinical remission was obtained in 7 (35%) and 9 (45%) patients, respectively; however, no significant discrepancy related to the LVEDV (*P* = 0.86), LVESV (*P*-value = 0.25), EF (*P*-value = 0.06), and GLS in standard parasternal short axis (*P* = 0.73), long axis [apical 2-chamber (*P*-value = 0.61), apical 3-chamber (*P*-value = 0.15), and apical 4-chamber (*P*-value = 0.19) views] was observed before and after the intervention. Furthermore, no statistically significant correlation between disease activity and cardiac function was found, neither before nor after ADA administration.

**Conclusion:**

The present perusal found no deterioration in left ventricular function indexes with ADA intervention among patients with IBD without cardiac ailment. Thus, prescribing the anti-TNF to alleviate the inflammation can be carried out with less concern about cardiac consequences and considering other adverse traces in the target group.

## 1. Introduction

Inflammatory bowel disease (IBD) is established by an inappropriate immune response that disarranges gastrointestinal hemostasis (1). Genetic vulnerability accompanied by environmental and immunological underlying mechanisms provokes this awkward immune response within the intestinal texture (2). Crohn's disease (CD) and ulcerative colitis (UC) constitute the main clinical manifestations of this chronic recurrent ailment, and they are predominantly specified with periods of flare and remission (3, 4). Inflammatory bases lead to a simultaneous flourishing of cardiovascular complications with IBD. Released cytokines, including tumor necrosis factor-α (TNF-α), disrupt endothelial function and consequence in arterial stiffness and atherosclerosis (5). Cardiac overpressure to conquer vascular rigidity results in heart failure over a long time (6, 7). Therefore, abundant evidence illuminated the TNF-α antagonism profitability in cardiac performance (8, 9). However, other studies revealed the role of anti-TNF in muscular contractile decrement (10, 11) and declared the conclusions against the previous hypothesis (12, 13). Thus, the controversy about anti-TNF usefulness needs to be tested. Infliximab and adalimumab (ADA) are the most extensive anti-TNF-α classes used (14). In addition, as Berg et al. declared, there is limited proof confirming anti-TNF impact in UC compared with CD (15). Global longitudinal strain (GLS) has been displayed as the cardiac screener that distinguishes microvascular perfusion disturbances before the traditional echocardiographic indicators. Therefore, for the early risk stratification of cardiac function, GLS should be measured besides other customary echocardiographic elements (16, 17).

Hence, this study aimed to appraise short-term anti-TNF relics on cardiac function through gauging echocardiography indexes in immunosuppressant refractory UC patients without cardiac ailment. Moreover, as a subsidiary aim, the association of disease activity and cardiac function before and after the pharmaceutical intervention was explored.

## 2. Materials and methods

This prospective observational cohort study was conducted from December 2020 to March 2022 in Taleghani Hospital (the main tertiary gastrointestinal referral center), Tehran, Iran. International guidelines on the clinical investigation from the World Medical Association's Declaration of Helsinki were preserved during the study; the ethics committee of Shahid Beheshti University approved the study protocol.

The study population consisted of all referred patients with a definite diagnosis (histopathological and endoscopic confirmation at least 3 months ago) of UC to the outpatient gastrointestinal clinic of Taleghani hospital. The required sample size, considering alpha-error: 5%, beta-error: 10%, mean difference: 0.5 [according to the adjusted statistics of similar investigations (18, 19)], and standard deviation (SD) change: 0.65, was calculated as 20 patients. They were included based on providing written informed consent and owning the severe form of active disease, which did not dampen with immunosuppressant (e.g., corticosteroids, azathioprine, and 6-mercaptopurine) therapy and, therefore, have been a candidate for biological treatment. Mayo score, a range of 0–12 points, constitutes endoscopic assessment, physician's self-evaluation, stool frequency, and rectal bleeding; it categorizes the subjects' disease activity with 0–1 score as remission, 2–4 as mild, 5–6 as moderate, and ≥7 as severe (20). Participants' enrollment with the severe uncontrolled inflammatory state was performed based on the condition of posing the Mayo score of 7 or more. The exclusion was applied for the patients with previous cardiac ailments, prior related surgical intervention, history of taking anti-TNF or anti-interleukin (IL) agents within the past 6 months, diabetes mellitus (DM), hypertension (HTN), hyperlipidemia (HLP), valvular heart disease, family history of atherosclerotic disease or coronary artery disorders at a young age, smoking, obesity, renal dysfunction, and ejection fraction (EF) <50%.

Data on age, sex, medical history, family, habitual, and drug history were recorded at the baseline. Moreover, characteristics related to UC were obtained, including the disease extent and duration (from diagnosis to the beginning of biological therapy). The Montreal classification was used to specify the disease extension and it involves three levels (21). L1 refers to ulcerative proctitis, in which the disease is limited to the rectum. L2 refers to left-side UC, in which inflammation is limited to the portion of the colon distal to the splenic flexure. L3 mentions pancolitis, in which the entire colon is involved.

Primary transthoracic echocardiography (TTE) was carried out before biological regimen allocation by an expert echocardiologist, unaware of participants' demographic information, *via* Siemens ACUSON SC2000 PRIME Ultrasound machine, where the measurement technique was the most accurate method available. EF, left ventricular end-diastolic volume (LVEDV), and left ventricular end-systolic volume (LVESV) were the indexes that were calculated at first. Then, two-dimensional speckle tracking echocardiography was performed to compute the left ventricular GLS in the standard parasternal short axis from mid-ventricular level, two-, three-, and four-chamber apical long axes. Images were captured during the breathing hold and in three continuous beats. LV has been divided into 16 segments to compute the amount of strain, and the average of segmental peak systolic longitudinal strain values is reported as the GLS (18). Subsequently, ADA was applied to the target population as a pharmaceutical intervention according to the protocol. In the induction phase, it is prescribed as 160 mg subcutaneous (SC), either as four injections of 40 mg on day 1 or as two injections of 40 mg daily on two consecutive days, then 80 mg SC 2 weeks later (day 15). The maintenance phase begins on the fourth week (day 29) with the 40 mg SC injection. It continues every 2 weeks based on the condition of observing any remission response within 8 weeks of therapy (22). At the end of the follow-up (3 months), participants were invited to undergo the echocardiography indexes rechecking and comparing the alteration *via* statistical methods. The number and percentage of intervention responders (endoscopic and clinical) were specified, and analytic analysis based on the type of response was accomplished. A partial Mayo score of two or fewer was considered the clinical remission, while a clinical response was defined as a decrease of two or more points. Diminution of the endoscopic Mayo score to one or null was defined as endoscopic remission. Furthermore, the association of disease activity and cardiac function, whether before or after the intervention, was evaluated by correlation testing between partial Mayo score and echocardiographic indexes.

Data were analyzed *via* the SPSS statistical software package version 24.0. The results were expressed as mean ± SD for quantitative variables and as a percentage for qualitative variables. The normality of variables was tested by Kolmogorov–Smirnov analysis. The significant discrepancy of stratified variables was examined *via* the chi-square test (or Pearson's χ^2^ test); meanwhile, independent and paired sample *t*-tests were used to compare the continuous values with the normal distribution. Spearman's or Pearson's analysis, based on variable normality, was applied to evaluate the correlation of continuous variables. A *p*-value <0.05 was considered statistically significant.

## 3. Results

**Figure 1:**
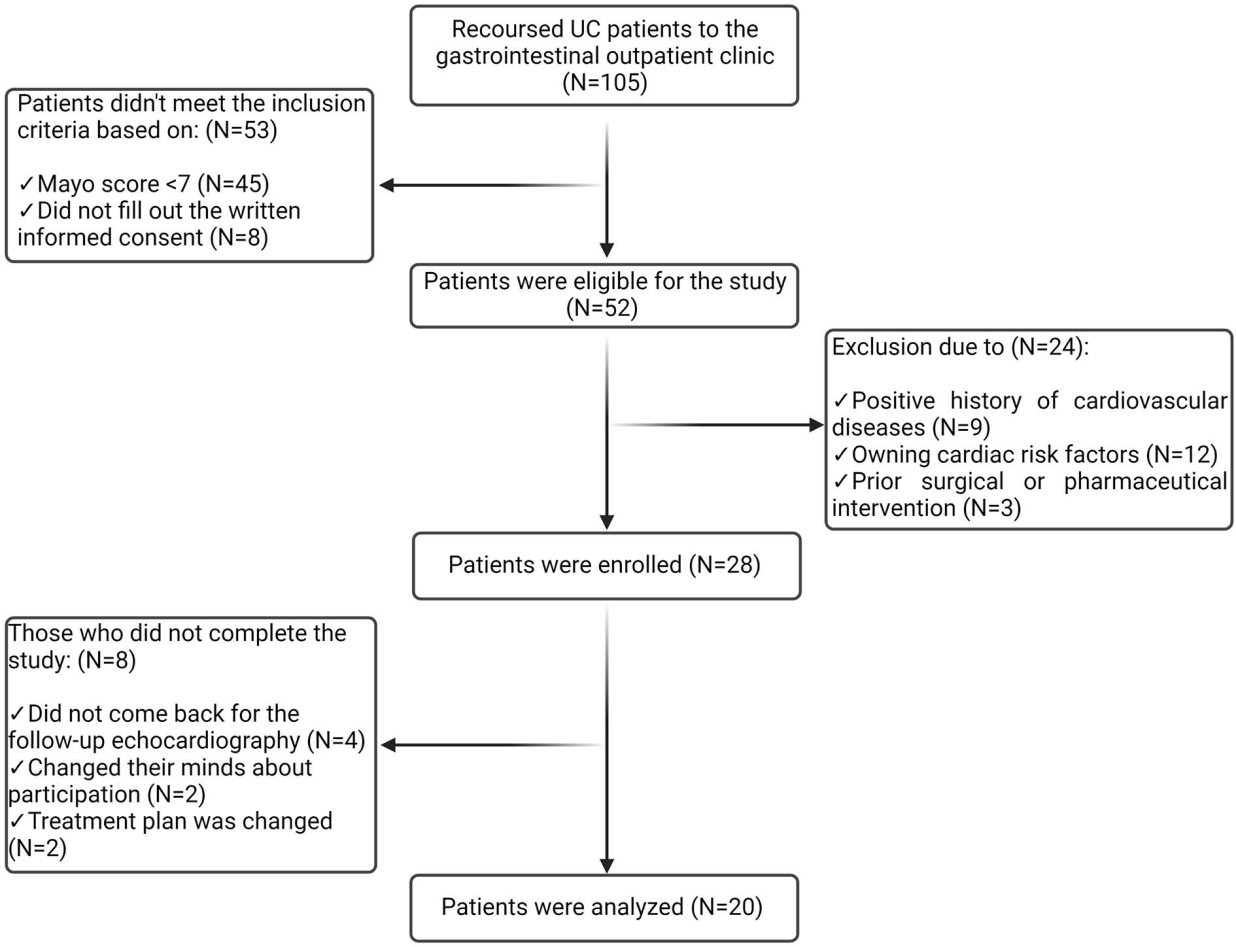
Diagram of the participants' selection process. Among 105 patients referred to the gastrointestinal outpatient clinic, 52 were eligible for the study. A total of 24 patients were excluded, and eight participants failed to complete the study. Thus, the data from 20 patients were analyzed.

A diagram of the participants' selection process is presented in Figure 1. Within the study, 105 cases were referred to the gastrointestinal outpatient clinic of Taleghani Hospital with a definite diagnosis of UC. Among them, 52 had a Mayo score of ≥ 7 and provided informed consent; thus, they were eligible for the study. A total of 24 patients were excluded after the primary assessment due to the following reasons: positive history of cardiovascular diseases (CVDs) (*n* = 9), owning cardiac risk factors including obesity, smoking, DM, HTN, HLP, and positive family history (*n* = 12), and prior surgical or pharmaceutical (anti-TNF or anti-IL) intervention (*n* = 3). Of the 28 participants who were enrolled in the study, eight patients did not complete the study as they missed the follow-up echocardiography (*n* = 4) and changed their minds about participation (*n* = 2) and the treatment plan (*n* = 2); thus, the data of 20 patients were analyzed.

The study consists of 13 (65%) men and 7 (35%) women, with a mean age of 40.38 ± 16.07 years and 32.71 ± 6.53 years, respectively. In these patients, UC mainly extended to the rectum (Montreal class I = 45%) and existed for an average of 3.25 years (Table 1).

**Table 1:** Descriptive and analytical analyses of demographic data before the intervention.

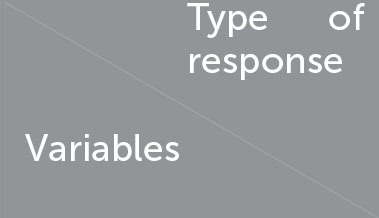	**All patients**	**Endoscopic status subclassification**		P^*^	**Clinical status subclassification**		P^*^
		**Remitted** **7 (35%)**	**Not-remitted** **13 (65%)**		**Remitted** **9 (45%)**	**Not-remitted** **11 (55%)**	
**Age (year)**	36.5 ± 11.3	38.0 ± 14.9	37.5 ± 14.2	0.947	41.0 ± 15.3	35.0 ± 13.1	0.358
**Gender %**							
Men	13 (65%)	4 (43%)	9 (69%)	0.651	5 (56%)	8 (73%)	0.423
Women	7 (35%)	3 (57%)	4 (31%)		4 (44%)	3 (27%)	
Montreal^**^							
L1 %	9 (45%)	6 (86%)	3 (23%)	**0.011**	8 (89%)	1 (9%)	**0.001**
L2 %	7 (35%)	1 (14%)	6 (46%)		1 (11%)	6 (55%)	
L3 %	4 (20%)	0 (0%)	4 (31%)		0 (0%)	4 (36%)	
Duration^***^	3.25 ± 2.4	1.57 ± 0.53	4.15 ± 2.54	**0.017**	1.78 ± 0.97	4.45 ± 2.58	**0.009**

* P refers to the P-value, the statistical significance of chi-square or independent samples T-test.

** In Montreal classification: L1 refers to ulcerative proctitis, L2 indicates the left-side UC, and L3 mentions pancolitis.

*** Disease duration demonstrates the period (years) between diagnosis and intervention initiation. Values in bold indicate statistically significant results.

**Figure 2:**
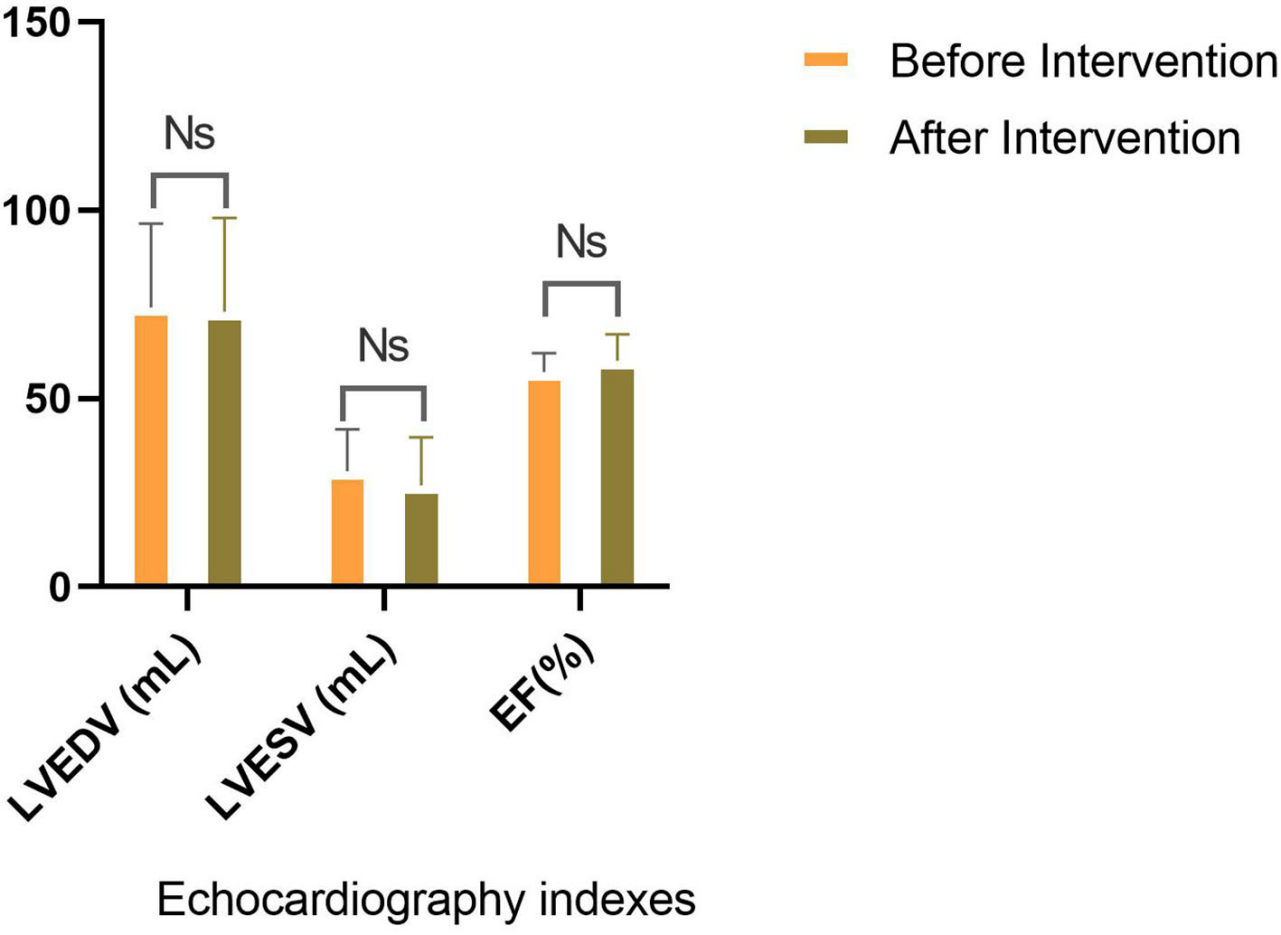
Comparison of traditional echocardiographic indices. No significant discrepancy for EF, LVESV, and LVEDV is seen before and after the intervention.

**Figure 3:**
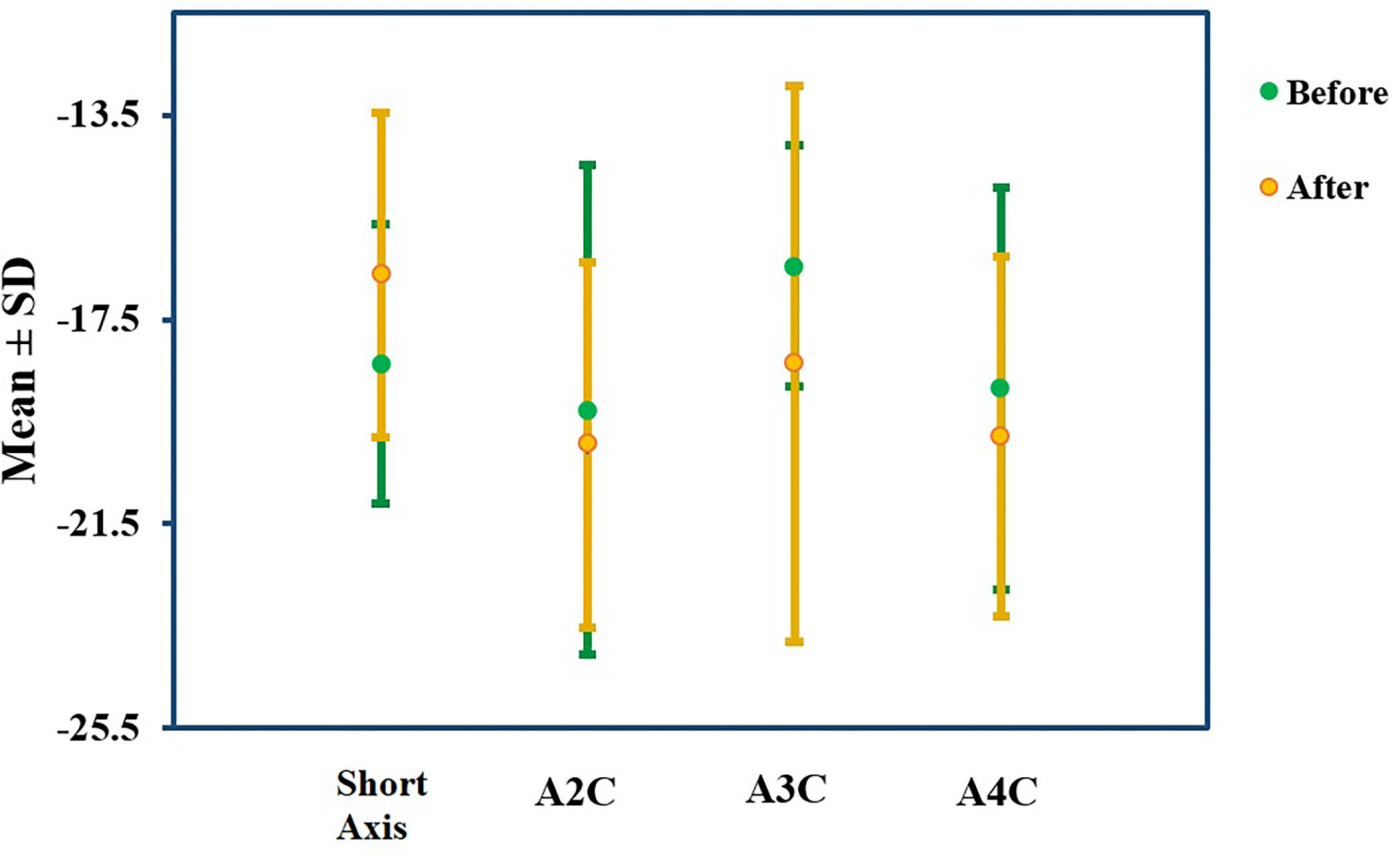
GLS percentage alteration. GLS did not change significantly in the standard parasternal short axis or two-, three-, and four-chamber apical long axes before and after the ADA administration.

The analysis illuminated that ADA has significantly reduced Mayo score, whether total (8.55 ± 1.23 vs. 5.00 ± 2.38) or endoscopic (2.65 ± 0.48 vs. 1.75 ± 1.02) or partial score (5.90 ± 1.21 vs. 3.25 ± 1.48) (Table 2). Subsequently, endoscopic remission, clinical remission, and clinical response were obtained in 7 (35%), 9 (45%), and 13 (65%) patients, respectively. The pharmaceutical intervention significantly controlled inflammation (C-reactive protein (CRP): 15.4 ± 4.50 mg/L vs. 1.31 ± 1.17 mg/L) and did not affect the renal system [blood urea nitrogen (BUN): 12.8 ± 4.12 mg/dl vs. 13.0 ± 2.83 mg/dl and creatinine 0.93 ± 0.21 mg/dl vs. 0.97 ± 0.17 mg/dl]. Mild elevation in liver enzymes (serum glutamic oxaloacetic transaminase: 21.0 ± 11.2 IU/L vs. 24.4 ± 11.2 IU/L, serum glutamic pyruvic transaminase: 26.2 ± 11.5 IU/L vs. 29.5 ± 13.1 IU/L, and alkaline phosphatase: 105 ± 26.0 IU/L vs. 111 ± 4.58 IU/L) was asymptomatic and was not presumed clinically significant. Alteration means values, before and after the ADA administration, related to the LVEDV (74.13 vs. 73.06 ml, *P*-value = 0.86), LVESV (30.66 vs. 26.93 ml, *P*-value = 0.25), and LVEF (57% vs. 60%, *P*-value = 0.06) were not significant (Figure 2). Similar to other indexes, GLS did not change significantly with intervention either in the standard parasternal short axis (−18.36 ± 2.73 vs. −18.63 ± 3.17, *P* = 0.73) or in long axis [apical 2-chamber (−19.26 ± 4.79 vs. −19.95 ± 3.57, *P*-value = 0.61), apical 3-chamber (−16.45 ± 2.37 vs. −18.35 ± 5.44, *P*-value = 0.15), and apical 4-chamber (−18.85 ± 3.93 vs. −19.80 ± 3.52, *P*-value = 0.19) views] (Figure 3).

**Table 2:** Descriptive and analytical analyses of clinical data based on the intervention stage.

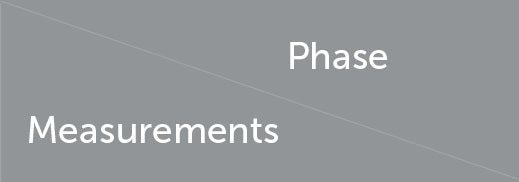	**Before intervention** **(mean ± SD or %)**	**After intervention** **(mean ± SD or %)**	*P* ^**^
**Mayo score**			
Partial score	5.90 ± 1.21	3.25 ± 1.48	**0.001**
Endoscopic sub-score	2.65 ± 0.48	1.75 ± 1.02	**0.001**
Total score	8.55 ± 1.23	5.00 ± 2.38	**0.001**
**Type of response**			
Endoscopic remission	–	35%	
Clinical remission	–	45%	
Clinical response	–	65%	
**Laboratory test**			
BUN (mg/dL)	12.8 ± 4.12	13.0 ± 2.83	0.606
Creatinine (mg/dL)	0.93 ± 0.21	0.97 ± 0.17	0.257
SGOT (IU/L)^*^	21.0 ± 11.2	24.4 ± 11.2	**0.015**
SGPT (IU/L)^*^	26.2 ± 11.5	29.5 ± 13.1	**0.004**
ALP (IU/L)^*^	105 ± 26.0	111 ± 4.58	**0.001**
CRP (mg/L)^*^	15.4 ± 4.50	1.31 ± 1.17	**0.001**
**Echocardiographic indices**			
LVEDV (mL)^*^	74.13 ± 22.30	73.06 ± 24.82	0.86
LVESV (mL)^*^	30.66 ± 11.14	26.93 ± 12.77	0.25
EF (%)^*^	57 ± 5	60 ± 7	0.06
**GLS**			
Standard short axis view	−18.36 ± 2.73	−18.63 ± 3.17	0.73
A2C view^*^	−19.26 ± 4.79	−19.95 ± 3.57	0.61
A3C view^*^	−16.45 ± 2.37	−18.35 ± 5.44	0.15
A4C view^*^	−18.85 ± 3.93	−19.80 ± 3.52	0.19

* SGOT, Serum glutamic oxaloacetic transaminase; SGPT, Serum glutamic pyruvic transaminase; ALP, Alkaline phosphatase; CRP, C-reactive protein; LVEDV, left ventricular end-diastolic volume; LVESV, left ventricular end-systolic volume; EF, ejection fraction; GLS, global longitudinal strain; A2C, apical two-chamber; A3C, apical three-chamber; A4C, apical four-chamber.

** P refers to the P-value, the statistical significance of paired sample T-test. Values in bold indicate statistically significant results.

Participants were grouped based on their type of response to the intervention, and data on demographic and clinical variables were compared among them (Table 3). Remitted cases (whether endoscopic or clinical) significantly possessed a shorter disease duration (endoscopic: 1.57 ± 0.53 years vs. 4.15 ± 2.54 years and clinical: 1.78 ± 0.97 years vs. 4.45 ± 2.58 years) and Montreal class I (endoscopic: 86 vs. 23% and clinical: 89 vs. 9%) at the baseline (Table 1). ADA was prescribed in the identical protocol for both groups; however, remitted subclasses owned higher anti-TNF concentration (endoscopic: 10.5 ± 1.71 μg/ml vs. 6.62 ± 0.91 μg/ml and clinical: 9.86 ± 2.03 μg/ml vs. 6.47 ± 0.90 μg/ml), lower level of CRP (endoscopic: 0.76 ± 0.06 mg/l vs. 1.60 ± 1.38 mg/l), and Mayo score (total score in endoscopic subclasses: 2.29 ± 0.95 vs. 6.46 ± 1.39 and in clinical subclasses: 2.67 ± 1.11 vs. 6.91 ± 0.94). In contrast, there is no significant discrepancy between the responders and non-responders in the laboratory tests and echocardiography indexes. In addition, no statistically significant correlation between disease activity and cardiac function was found, neither before the ADA nor after its administration (Table 4).

**Table 3:** Analytical analyses of clinical data based on the type of response after the intervention.

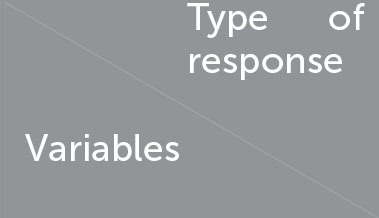	**Endoscopic status subclassification**		P^**^	**Clinical status subclassification**		P^**^
	**Remitted** **7 (35%)**	**Not-remitted** **13 (65%)**		**Remitted** **9 (45%)**	**Not-remitted** **11 (55%)**	
**Mayo score**						
Partial score	1.71 ± 0.48	4.08 ± 1.11	**0.001**	1.78 ± 0.44	4.45 ± 0.68	**0.001**
Endoscopic sub-score	0.57 ± 0.53	2.38 ± 0.50	**0.001**	0.89 ± 0.78	2.45 ± 0.52	**0.001**
Total score	2.29 ± 0.95	6.46 ± 1.39	**0.001**	2.67 ± 1.11	6.91 ± 0.94	**0.001**
**Anti-TNF** **α** **concentration (μg/mL)**	10.5 ± 1.71	6.62 ± 0.91	**0.001**	9.86 ± 2.03	6.47 ± 0.90	**0.001**
**Laboratory test**						
BUN (mg/dL)	12.1 ± 2.34	13.5 ± 3.04	0.307	12.8 ± 2.80	13.1 ± 2.99	0.825
Cr (mg/dL)^*^	1.02 ± 0.21	0.94 ± 0.15	0.335	0.98 ± 0.21	0.96 ± 0.15	0.761
SGOT (IU/L)^*^	25.8 ± 13.6	23.6 ± 10.2	0.693	27.7 ± 14.1	21.7 ± 7.91	0.242
SGPT (IU/L)^*^	31.5 ± 14.7	28.3 ± 12.6	0.618	34.7 ± 18.0	25.1 ± 4.62	0.155
ALP (IU/L)^*^	110 ± 11.5	111 ± 37.5	0.951	109 ± 11.2	113 ± 40.7	0.735
CRP (mg/L)^*^	0.76 ± 0.06	1.60 ± 1.38	**0.048**	0.82 ± 0.13	1.70 ± 1.48	0.079
**Echocardiographic indices**						
LVEDV (mL)^*^	74.87 ± 24.9	72.08 ± 25.7	0.818	74.01 ± 29.7	72.28 ± 21.5	0.882
LVESV (mL)^*^	25.20 ± 9.60	27.83 ± 14.4	0.681	27.22 ± 15.0	26.70 ± 11.3	0.930
EF (%)^*^	60.43 ± 5.76	60.69 ± 7.52	0.937	60.33 ± 5.59	60.82 ± 7.92	0.879
**GLS**						
SSA view^*^	−19.35 ± 2.53	−18.23 ± 3.50	0.467	−18.94 ± 2.35	−18.37 ± 3.81	0.703
A2C view^*^	−21.28 ± 4.65	−19.23 ± 2.79	0.315	−20.51 ± 4.33	−19.49 ± 2.96	0.542
A3C view^*^	−18.30 ± 5.94	−18.38 ± 5.41	0.975	−17.72 ± 5.31	−18.87 ± 5.75	0.650
A4C view^*^	−19.49 ± 4.00	−19.97 ± 3.39	0.780	−19.37 ± 3.51	−20.15 ± 3.66	0.637

* Cr, Creatinine; SGOT, Serum glutamic oxaloacetic transaminase; SGPT, Serum glutamic pyruvic transaminase; ALP, Alkaline phosphatase; CRP, C-reactive protein; LVEDV, left ventricular end-diastolic volume; LVESV, left ventricular end-systolic volume; EF, ejection fraction; GLS, global longitudinal strain; SSA, Standard short axis; A2C, apical two-chamber; A3C, apical three-chamber; A4C, apical four-chamber.

** P refers to the P-value, the statistical significance of independent samples T-test. Values in bold indicate statistically significant results.

**Table 4:** Pearson's correlation coefficient of disease activity and cardiac function.

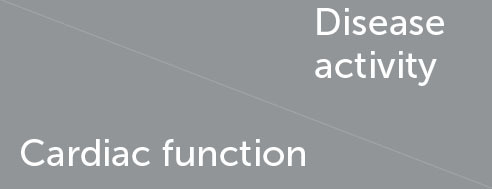	**Partial Mayo score**			
	**Before intervention**		**After intervention**	
	**r-coefficient**	***P*** **-value**	**r-coefficient**	***P*** **-value**
**Echocardiographic indexes**				
LVEDV (mL)	−0.048	0.842	−0.013	0.956
LVESV (mL)	−0.095	0.690	−0.144	0.544
EF (%)	0.011	0.963	0.099	0.679
**GLS**				
SSA view	−0.108	0.651	−0.084	0.725
A2C view	−0.056	0.814	−0.385	0.094
A3C view	−0.061	0.800	−0.094	0.693
A4C view	−0.014	0.955	−0.221	0.349

LVEDV, left ventricular end-diastolic volume; LVESV, left ventricular end-systolic volume; EF, ejection fraction; GLS, global longitudinal strain; SSA, Standard short axis; A2C, apical two-chamber; A3C, apical three-chamber; A4C, apical four-chamber.

## 4. Discussion

### 4.1. IBD, inflammation, and CVDs

**Figure 4:**
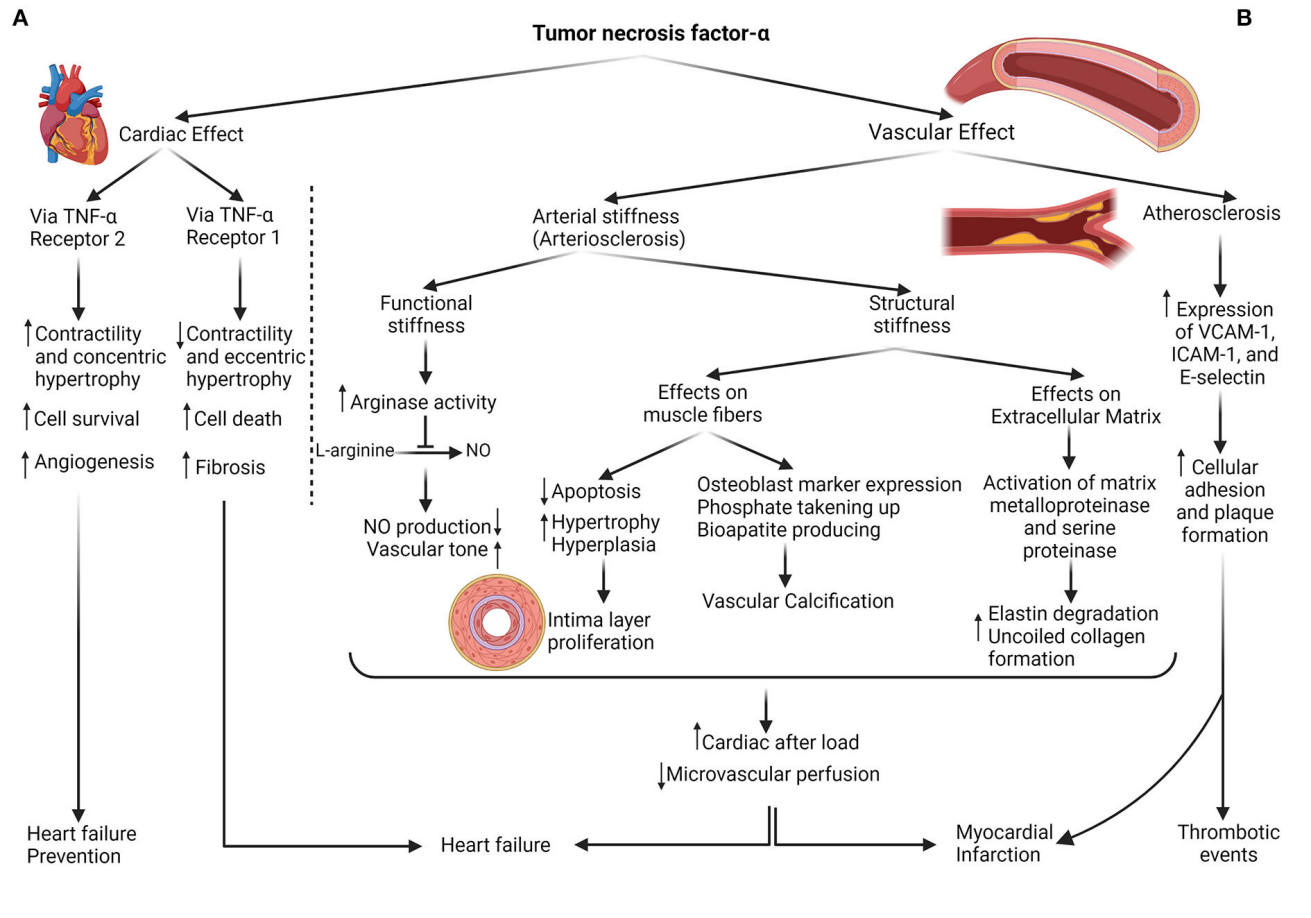
The dual role of TNF-α in cardiovascular consequences. **(A)** Cardiac effects: TNF-α can enhance contractility, concentric hypertrophy, cell survival, and angiogenesis by instigating type 2 TNF-α receptors on heart tissue, protective mechanisms against heart failure development; also, it can either exert its role with type 1 TNF-α receptor provocation in the contractility decrement, cell death, and fibrosis. **(B)** Vascular effects: production of NO decreases with the impact of TNF-α on the Arginase enzyme, resulting in vascular hypertonicity. In addition, with the influence of this cytokine on muscle fibers and extracellular matrix, vascular hypertrophy, and calcification, accompanied by less flexible ECM, are shaped. As a result of vascular stiffness (structural and functional), cardiac afterload raises, and microvascular perfusion diminishes. The other vascular sequel of TNF-α is atherosclerosis or plaque formation which is facilitated by the expression of cellular adhesion molecules such as VCAM, ICAM, and E-selectin. Both heart and vessels adverse outcomes contribute to cardiovascular event predisposition.

Forecasted cardiovascular morbidity and mortality rates of patients with IBD were wrong. Unlike the low prevalence of traditional CVDs' hazard factors, such as BMI and lipid composition of this group in collation with public society (23), more cardiac consequences, including heart failure, myocardial infarction, and thrombosis events, were observed (24). However, recent investigations have clarified that more prevalent cardiac risk factors in patients with IBD comprising diabetes, hypercholesterolemia, HTN, and physical inactivity predisposed them to further CVDs (25). The assertion of the shared territory between IBD and CVDs was postulated based on different concepts. Increasing the intestinal epithelial permeability arising from dysbiosis within the IBD pathogenesis exposes the local leukocytes to the microbiome. It develops an inappropriate immune reaction by releasing inflammatory cytokines, specifically TNF-α (26). Nitric oxide (NO), endothelin, von Willebrand factor, prostacyclin, and cellular adhesion molecules are fabricated mediators by the endothelium that regulate vascular hemostasis, including cellular adhesion, immune cell diapedesis inhibition, and a vasodilation-constriction balance (27). Released inflammatory cytokines alter these mediators, which is named endothelial dysfunction (Figure 4) because, without these factors, the endothelium cannot exert its role in adjusting vascular hemostasis (28). TNF-α augments the arginase activity (an enzyme that consumes the L-arginine substrate in endothelial cells). Thus, it decreases the L-arginine catalyzation to produce the NO in another pathway. Diminished NO level leads to enhanced vascular tone and arterial stiffness. This reversible process is known as functional stiffness (29, 30). In addition, irreversible structural stiffness occurs within the three steps, simultaneously with functional stiffness. In the first step, TNF-α and IL-1 lead to apoptosis decrement, hypertrophy, and hyperplasia (intima layer proliferation) in smooth muscle fibers (31). With the expression of the osteoblast marker on smooth muscle cells by the effect of these inflammatory cytokines, phosphate is taken up, and bioapatite is produced, resulting in vascular calcification (32). In the last stage, TNF-α provokes and activates matrix metalloproteinase and serine proteinase, resulting in elastin fiber degradation and uncoiled collagen formation (33). Elevated expression of VCAM-1, ICAM-1, and E-selectin (crucial elements for T-cell and monocyte adherence) is recognized in patients with IBD exposed to inflammation. This adhesion provides the basis for subsequent atherosclerosis (34). In summary, arteriosclerosis and atherosclerosis (two different concepts) exist as a bridge between IBD inflammation and CVDs.

#### 4.1.1. Arterial stiffness or arteriosclerosis

Arterial stiffness has been introduced as a crucial mediator between IBD and CVDs. Pooled results of documents in one of the most recent meta-analyses indicated that both UC and CD categories are correlated with arterial stiffness (7). The role of systemic inflammation, independent of atherosclerosis, in inducing arterial stiffness has been validated by investigating other inflammatory disorders such as rheumatoid arthritis (35) and Kawasaki disease (36). Vascular stiffness modifies both components of systemic blood pressure (causing the wide pulse pressure). Systolic blood pressure (afterload) rises, and LV is forced to work more intensely, resulting in heart failure in the long term. Diastolic pressure is alleviated; therefore, microvascular perfusion on various organs disrupts. Both cytokine traces and LV overwork lead to hypertrophy and oxygen requirement augmentation. Also, the hypertrophied cardiac muscle relaxes slowly; hence, LVED volume diminishes, and a lower amount of blood to pump exists. Altogether, perfusion disruption, reduced LVED volume, and cellular demand increment predispose the individual to ischemic events such as myocardial infarction and stroke (6).

#### 4.1.2. Atherosclerosis and plaque formation

Distinct pathways for thrombus generation have been described. First, increased cellular attachment to the vascular inner surface, in addition to the provoked hypercoagulable state in the setting of IBD (37), induces or accelerates atherosclerosis and ultimately results in a higher risk of thrombosis events (38). Arvanitakis et al., in a recent meta-analysis, exposing the 2-fold increased risk of venous thromboembolism in patients with IBD, emphasized prophylaxis and primary prevention importance (39). Second, a thrombus formation interceded by atrial fibrillation during the active phase of IBD was illustrated by Kristensen et al. (40). This hypothesis was confirmed when Dogan et al. demonstrated that P-wave dispersion (an indicator for atrial fibrillation) is higher in patients with IBD (41).

#### 4.1.3. Anti-TNF dilemma

Experiments were designed to measure the potency of immunomodulatory interventions such as anti-TNF for diminishing arterial stiffness. Zanoli et al., appraising the pulse wave velocity (PWV) alteration during the 3.5 years of follow-up, declared the benefits of anti-TNF and steroids in 7 and 11 subjects with IBD, respectively, compared to 30 matched control subjects (42). Angel et al. revealed that anti-TNF ameliorates the PWV and arterial stiffness and alleviates the carotid intima-media thickness progression as the prognostic marker for major advance cardiac events (8). In contrast, certain substantial evidence has emerged on the disadvantages of anti-TNF. Grillo et al. observed the heart failure symptoms 30 days after anti-TNF (infliximab) commencement and declared that short-term TNF-α antagonism did not demonstrate a benefit (12). Kwon et al. recorded the heart failure signs after an average of 3.5 months, anti-TNF therapy in 47 patients with rheumatoid arthritis, psoriatic arthritis, and CD; 82% of them represented new-onset cardiac failure (13). Lecour et al. exhibited the cardiac protection of TNF-α *via* sphingolipid signaling intermediates, so TNF-α deprives this utility (43). Cacciapaglia et al. clarified the dual role of anti-TNF-α therapy that can either possess toxic cardiac effect prevention or interfere with the beneficial preconditioning effects of anti-TNF-α (10). Then, Besse et al. claimed that exerting the TNF-α role depends on its receptor. Pathways related to TNF-receptor 1 enhance angiogenesis, concentric hypertrophy, and contractile increment; meanwhile, TNF-receptor 2 instigation leads to vascular fibrosis, eccentric hypertrophy, and contractile decrement (Figure 4) (11). In addition, side effects, including opportunistic infections, lymphoma, malignancies progression, and skin damage, should be deliberated when administering anti-TNF-α (44). Therefore, a considerable controversy surrounding anti-TNF use emerged based on its dual role and has become the reason for the current research.

### 4.2. UC, ADA, and GLS

There are more reports on anti-TNF outcomes in patients with CD, and the necessity of such evaluation in patients with UC is felt (15, 19). Anti-TNF, including certolizumab, ADA, and infliximab, is prescribed in intolerant or refractory IBD cases to immunosuppressants (e.g., corticosteroids) (45). ADA has been indicated to be more efficient with the occurrence of lower complications than other classes (46); hence, it has been chosen as the most extensive TNF-α antagonism used for the current investigation. Cardiac function can be screened through diverse instruments; nevertheless, a new non-invasive method is strain assessment. Due to the high sensitivity of tissue Doppler imaging and D2 base strain, the procedure is suitable for early detection of myocardial dysfunction and sub-endothelial damage before EF alteration (16). Thus, GLS seems more beneficial than conventional echocardiography for functional appraisement. Overall, the effect of ADA (as the anti-TNF) on GLS (as the main cardiac function scale) and other indicators in immunosuppressant refractory UC patients (as an IBD subtype) was scrutinized.

The present perusal found neither worsening nor improvement with anti-TNF intervention. Vizzardi et al. tracked the GLS alteration induced by anti-TNFs (ADA in 5 cases and etanercept and infliximab in 4 cases) on patients with rheumatoid arthritis without cardiac ailments for 1 year and observed no discrepancy. Their conclusion is aligned with the current perusal; however, they scrutinized different groups with the lower subjects in more extended follow-ups (47). Triantafyllou et al. examined 45 patients with CD and 15 patients with UC at the baseline and four months after anti-TNF therapy (ADA or infliximab) and illuminated a significant enhancement in GLS. Although the methodology is similar to the present investigation (the same group, follow-up period, intervention, and cardiac progression factor), their opposed results may be justified by the smaller sample size (19). Costantino et al. declared that anti-TNF biological agents (infliximab, ADA, or vedolizumab) positively affect GLS (as a cardiac risk indicator). A contrary conclusion may be due to their smaller sample size (16 patients with IBD) or additional follow-up time (6 months after intervention) (48).

### 4.3. Disease activity and cardiac function

One of the most prevalent manifestations of UC is diarrhea which is correlated with the disease activity index; the higher the severity of UC, the greater the amount of diarrhea and fluid lost in the body. Severe dehydration, if not compensated, leads to a decrement in cardiac venous return and preload; based on Frank–Starling law, this can result in contractile attenuation (49). In addition, inflammation can affect colon ionic (e.g., sodium or potassium necessary for cardiac hemostasis) absorption, which subsequently appears in the cardiac contractility change. Less contraction ability results in a lower muscle fiber strain pattern; thus, as Cicin et al. (18) declared, disease activity status (Mayo score) in patients with UC and GLS are negatively correlated. However, this project failed to demonstrate such a correlation, whether before or after the intervention. This may be due to different study populations, all cases with the severe form of the disease. In addition, laboratory tests indicated that these participants' dehydration had been attenuated, with a compensatory mechanism such as oral hydration.

### 4.4. Strengths and limitations

A general verdict about the effect of anti-TNF therapy on heart function in IBD patients cannot be made because different subsets of settings (types of groups, interventions, and measurements) are involved. However, exclusive changes (limiting the anti-TNF to the ADA class, IBD patients to UC cases, and cardiac function to the GLS) deducted the cofounders' influence and made the study more accurate. Although this project achieved its aims, some limitations should be noted. Based on inclusion and exclusion criteria, the sample size was refined to a slightly smaller size so that a similar study with more prominent participants is recommended for a firm decision regarding anti-TNF utilization. More studies exhibit priming of the cardiac complications in a short period after anti-TNF (12, 13), which was the basis of the current perusal sketch; hence, a resemble inquiry with a long-term follow-up is suggested.

## 5. Conclusion

The present investigation explored the anti-TNF therapy consequences on heart performance by monitoring EF, LVESV, LVEDV, and GLS within the short-term (3 months) follow-up of immunosuppressant-resistant patients with UC. The absence of significant change in ventricular performance indicators demonstrated that anti-TNF can be utilized with less concern about cardiac consequences and more attention to the other adverse traces in the target group. Further studies on patients with moderate-to-severe decreased cardiac function to investigate the synergy of anti-TNF and heart failure are needed; likewise, other patients with autoimmune diseases, including CD, who are receiving ADA, should be considered.

## Data availability statement

The raw data supporting the conclusions of this article will be made available by the authors, without undue reservation.

## Ethics statement

The studies involving human participants were reviewed and approved by the Shahid Beheshti University Ethics Committee, Tehran, Iran. Written informed consent to participate in this study was provided by the participants' legal guardian/next of kin.

## Author contributions

Conception or design of the study: SS, MK, and HA. Acquisition of data: FJ, MM, HB, and MR. Analysis or interpretation of data: FS and MH. Drafting the article: MH, NE, and FS. Critical final revision: SB, SS, MH, and HA. All authors approved the final version to be published.
